# The impact of the COVID-19 pandemic on semen quality of uninfected men

**DOI:** 10.1186/s12610-022-00180-w

**Published:** 2023-03-09

**Authors:** Wenjun Zhang, Li Wang, Jiwei Sun, Linlin Cui, Haobo Zhang, Jingmei Hu

**Affiliations:** 1 Center for Reproductive Medicine, Cheeloo College of Medicine, Shandong University, 250012 Jinan, Shandong China; 2 Key laboratory of Reproductive Endocrinology of Ministry of Education, Shandong University, 250012 Jinan, Shandong China; 3 Shandong Key Laboratory of Reproductive Medicine, 250012 Jinan, Shandong China; 4 Shandong Provincial Clinical Research Center for Reproductive Health, 250012 Jinan, Shandong China; 5 National Research Center for Assisted Reproductive Technology and Reproductive Genetics, Shandong University, 250012 Jinan, Shandong China

**Keywords:** COVID-19, Semen quality, Sperm donor, Human sperm bank

## Abstract

**Background:**

Coronavirus disease 2019 (COVID-19) has spread rapidly worldwide since its discovery in December 2019. Research published since the COVID-19 outbreak has focused on whether semen quality and reproductive hormone levels are affected by COVID-19. However, there is limited evidence on semen quality of uninfected men. This study aimed to compare semen parameters among uninfected Chinese sperm donors before and after the COVID-19 pandemic to determine the impact of the COVID-19 pandemic-related stress and lifestyle changes on uninfected men.

**Results:**

All semen parameters were non-significant except semen volume. The average age of sperm donors was higher after the COVID-19 (all *P* < 0.05). The average age of qualified sperm donors increased from 25.9 (SD: 5.3) to 27.6 (SD: 6.0) years. Before the COVID-19, 45.0% qualified sperm donors were students, but after the COVID-19, 52.9% were physical laborers (*P* < 0.05). The proportion of qualified sperm donors with a college education dropped from 80.8 to 64.4% after the COVID-19 (*P* < 0.05).

**Conclusion:**

Although the sociodemographic characteristics of sperm donors changed after the COVID-19 pandemic, no decline in semen quality was found. There is no concern about the quality of cryopreserved semen in human sperm banks after the COVID-19 pandemic.

## Résumé

**Contexte:**

La maladie due au Coronavirus 2019 (COVID-19) s’est propagée rapidement dans le monde entier depuis sa découverte en décembre 2019. Les recherches publiées depuis l’éclosion de la COVID-19 se sont concentrées sur la question de savoir si la qualité du sperme et les niveaux d’hormones reproductives étaient affectés par la COVID-19. Il existe, cependant, peu de preuves sur la qualité du sperme des hommes non infectés. Cette étude visait à comparer, avant et après la pandémie de COVID-19, les paramètres du sperme chez les donneurs de sperme Chinois non infectés, afin de déterminer l’impact du stress lié à la pandémie de COVID-19 et aux changements de mode de vie sur les hommes non infectés.

**Résultats:**

Toutes les valeurs des paramètres du sperme étaient non significatives, à l’exception du volume de sperme. L’âge moyen des donneurs de sperme était plus élevé après la COVID-19 (tous *p* < 0,05). L’âge moyen des donneurs de sperme admissibles est passé de 25,9 ans (ET : 5,3) à 27,6 ans (ET : 6,0). Avant la COVID-19, 45 % des donneurs de sperme admissibles étaient des étudiants, mais après la COVID-19, 53 % étaient des travailleurs physiques (*p* < 0,05). La proportion de donneurs de sperme admissibles ayant fait des études secondaires est passée de 80,8 % à 64,4 % après la COVID-19 (*p* < 0,05).

**Conclusion:**

Bien que les caractéristiques sociodémographiques des donneurs de sperme aient changé après la pandémie de COVID-19, aucune baisse de la qualité du sperme n’a été constatée. Il n’y a aucune préoccupation quant à la qualité du sperme cryoconservé dans les banques de sperme humain après la pandémie de COVID-19.

**Mots-clés:**

COVID-19, Qualité du Sperme, Donneur de Sperme, Banque de Sperme humain.

## Introduction

Since its discovery in December 2019, coronavirus disease 2019 (COVID-19), caused by severe acute respiratory syndrome coronavirus 2 (SARS-CoV-2), has rapidly spread worldwide, posing a major threat to the health of people. Health behaviors, stress levels, and financial security of the general population have been impacted by the COVID-19 pandemic [1–3]. SARS-CoV-2 binds to the angiotensin-converting enzyme 2 (ACE2) receptor in humans [4, 5], which is enriched in spermatogonia and Leydig and Sertoli cells [6]. These findings raise concerns regarding the effects of COVID-19 on male semen quality. Holtmann et al. [7] demonstrated a decline in the number of progressively motile spermatozoa, sperm concentration, and total sperm count in men with moderate infection. Gacci et al. [8] found that 11 out of 43 recovered patients demonstrated oligo-crypto-azoospermia. However, there is considerable uncertainty regarding the effects of COVID-19 on male fertility.

As the most widespread pandemic in this century, the COVID-19 pandemic has raised concerns about the reproductive health of uninfected men. After the COVID-19 outbreak, people experienced more emotional stress reactions, including anxiety and depression [9, 10]; more sitting time and screen time; and less physical activity [11]. The influence of psychological stress, lifestyle, and mobile phone use on semen quality has long been discussed [12–14]. However, there is limited evidence on semen quality of uninfected men during the COVID-19 pandemic. Therefore, this study was designed to investigate the impact of the COVID-19 pandemic-related stress and lifestyle changes on semen quality of uninfected men based on 1487 semen samples collected from the Shandong Human Sperm Bank of China.

## Materials and methods

### Study design and participants

**Fig. 1:**
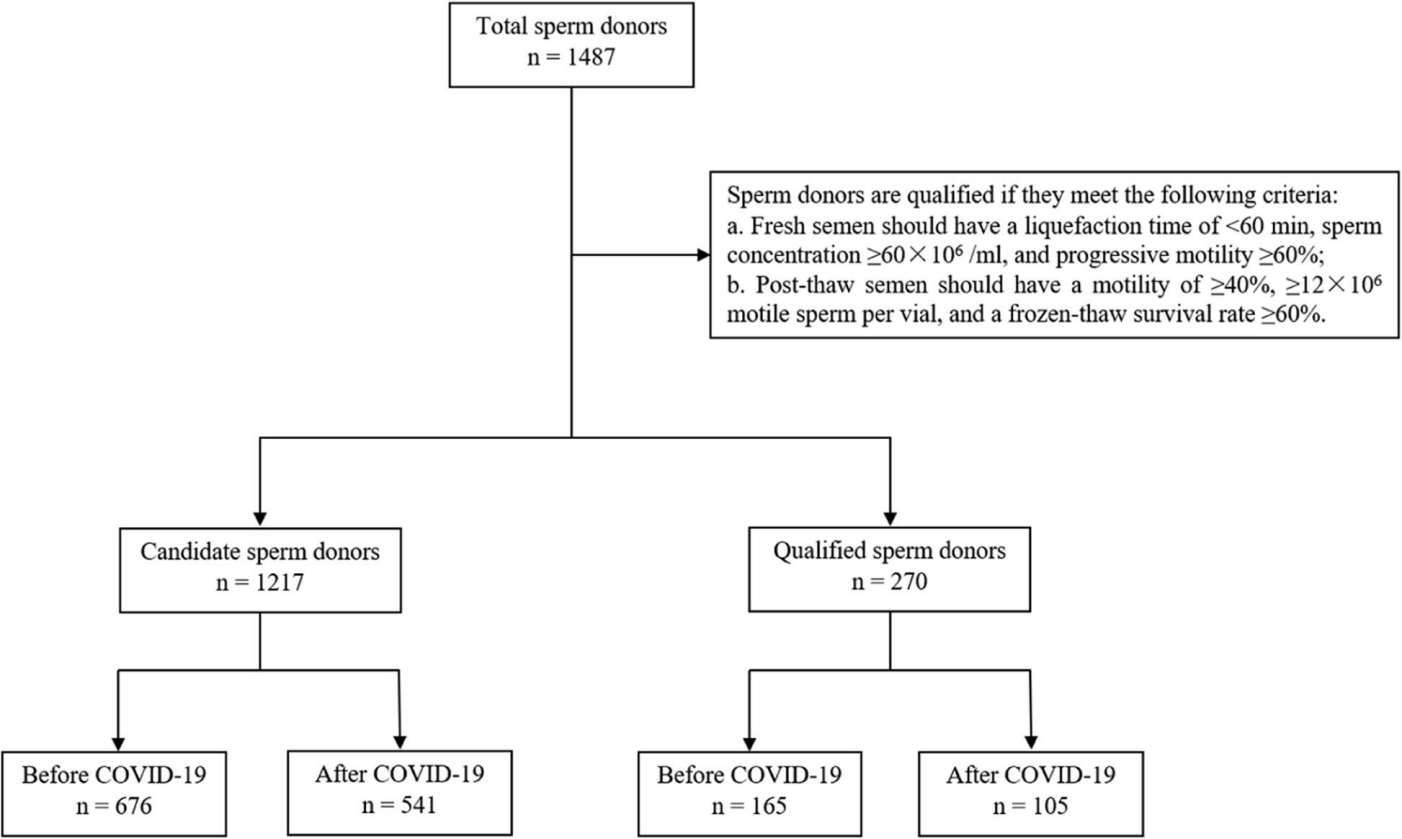
Flow diagram of sperm donor enrollment

This retrospective study of sperm donors was performed at the Shandong Human Sperm Bank of China from January to June 2019 and January to June 2020. The sociodemographic characteristics including age and abstinence time of all sperm donors; and body mass index (BMI), education level, and occupation of qualified sperm donors, were obtained from the database of the Shandong Human Sperm Bank. Sperm donors were strictly screened according to the standard guidelines issued in 2003 by China’s Ministry of Health. Individuals meeting the criteria for donors were qualified sperm donors (Fig. 1). After the outbreak of the COVID-19, the sperm donors were asked to complete the questionnaire for COVID-19 and show their travel codes, which can indicate the history of close physical contact or travel to high-risk areas, and the nasopharyngeal swab testing results, when they visited the sperm bank to ensure that they were not infected with SARS-CoV-2. All sperm donors signed informed consent allowing the use of their semen samples and clinical data in scientific research. The study was approved by the Reproductive Medicine Ethics Committee, Hospital for Reproductive Medicine Affiliated to Shandong University.

### Semen collection and analysis

The 1217 semen samples of candidate sperm donors and 270 samples of qualified sperm donors were included in the study. All semen samples were obtained by masturbation into a sterile plastic container after 2–7 days of abstinence. Semen analysis was performed according to the guidelines of the World Health Organization Fifth edition [15]. The semen samples were liquefied in a 37 °C water bath and analyzed within 1 h after collection. The semen parameters, including volume, sperm concentration, total sperm count, progressive motility, total motility, number of progressive motility, and normal sperm morphology were assessed. Semen volume was estimated by graduated pipettes. Sperm concentration and motility were assessed by the manual counting method with the Makler chamber. Total sperm count was calculated as sperm concentration multiplied by semen volume. During the research, quality control was conducted regularly in the laboratory with satisfactory results of CV within 10%.

### Statistical analysis

Normality of the data distribution was determined by the Shapiro-Wilk test. Student’s *t*-test and Mann-Whitney U test were used to describe differences in sociodemographic characteristics and semen parameters before and after the COVID-19 pandemic. Data were given as mean (standard deviation, SD) or median (interquartile range, IQR) for the results. Differences between categorical data were compared using the chi-square test or Fisher’s exact test. Natural-log transformations were performed on semen parameters to approximate the normality assumption. To eliminate the effects of covariates on qualified sperm donors, including age, education level, and occupation, we adjusted for covariates by multiple linear regression analysis. We used SPSS 24.0 to perform the statistical analysis. Two-sided *P* values < 0.05 were considered to be statistically significant.

## Results

The sociodemographic characteristics and semen parameters of candidate sperm donors are shown in Table 1. The average age of candidate sperm donors was 26.0 (SD: 5.2) years before the outbreak, while it was 26.7 (SD: 5.7) years after the outbreak (*P* < 0.05). After the outbreak, the median semen volume was 2.4 (IQR: 1.9) ml, compared with 2.0 (IQR: 1.6) ml before the outbreak (*P* < 0.05). There were no findings of significant differences in other semen parameters of candidate sperm donors before and after the COVID-19 (all *P* > 0.05).

**Table 1:** Comparison of sociodemographic characteristics and semen parameters of candidate sperm donors before and after the COVID-19 pandemic Age and abstinence time were presented as mean (SD) for the results. Semen parameters were presented as median (IQR) for the results. Differences between age and abstinence time were compared using Student’s *t*-test. Differences between semen parameters were compared using Mann-Whitney U test. *SD* standard deviation, *IQR* interquartile range

	Before COVID-19 (*n* = 676)	After COVID-19 (*n* = 541)	*P* value
**Age** (years) (mean, SD)	26.0 (5.2)	26.7 (5.7)	0.031
**Abstinence time** (days) (mean, SD)	4.9 (1.4)	4.8 (1.3)	0.065
**Semen parameters** (median, IQR)			
Volume (ml)	2.0 (1.6)	2.4 (1.9)	0.028
Sperm concentration (10^6^/ml)	31.0 (23.0)	30.0 (22.0)	0.964
Total sperm count (10^6^)	64.0 (79.4)	69.6 (88.8)	0.136
Progressive motility (%)	36.0 (15.0)	36.0 (17.0)	0.754
Total motility (%)	47.0 (16.8)	48.0 (18.5)	0.670
Number of progressive motility (10^6^)	21.0 (31.0)	24.18 (32.9)	0.221

Age and abstinence time were presented as mean (SD) for the results. Semen parameters were presented as median (IQR) for the results. Differences between age and abstinence time were compared using Student’s t-test. Differences between semen parameters were compared using Mann-Whitney U test. SD: standard deviation, IQR: interquartile range

The sociodemographic characteristics and semen parameters of qualified sperm donors are shown in Table 2. The average age of qualified sperm donors was 25.9 (SD: 5.3) years before the outbreak and 27.6 (SD: 6.0) years after the outbreak (*P* < 0.05). Significant differences in the distribution of education level and occupation were observed (all *P* < 0.05). The education level of qualified sperm donors was college or higher, accounting for 80.8% and 64.4% before and after the outbreak, respectively. Before the outbreak, 45.0% qualified donors were students and 22.5% were physical laborers. After the outbreak, 19.2% qualified donors were students and 52.9% were physical laborers. We did not find significant differences in volume, sperm concentration, total sperm count, progressive motility, total motility, number of progressive motility, and normal sperm morphology of qualified sperm donors before and after the COVID-19 (all *P* > 0.05).

**Table 2:** Comparison of sociodemographic characteristics and semen parameters of qualified sperm donors before and after the COVID-19 pandemic

	Before COVID-19 (*n* = 165)	After COVID-19 (*n* = 105)	*P* value
**Age** (years) (mean, SD)	25.9 (5.3)	27.6 (6.0)	0.018
**Abstinence time** (days) (mean, SD)	4.9 (1.4)	4.8 (1.4)	0.491
**BMI** (kg/m^2^) ^a^ (mean, SD)	23.4 (3.1)	23.9 (2.9)	0.234
**Education level**^**b**^			0.003
Less than college (%)	19.2	35.6	
College or higher (%)	80.8	64.4	
**Occupation**^c^			< 0.001
Mental labor (%)	29.8	25.0	
Physical labor (%)	22.5	52.9	
Unemployment (%)	2.6	2.9	
Student (%)	45.0	19.2	
**Semen parameters** (median, IQR)			
Volume (ml)	2.4 (1.3)	2.4 (1.2)	0.941
Sperm concentration (10^6^/ml)	60.0 (29.0)	56.0 (30.0)	0.153
Total sperm count (10^6^)	140.0 (99.6)	124.0 (111.8)	0.374
Progressive motility (%)	52.0 (14.0)	52.0 (10.0)	0.826
Total motility (%)	62.0 (12.0)	62.0 (8.0)	0.632
Number of progressive motility (10^6^)	69.1 (62.6)	65.5 (66.3)	0.668
Normal sperm morphology (%)	6.5 (3.5)	6.5 (2.5)	0.192

Age, abstinence time, and BMI were presented as mean (SD) for the results. Semen parameters were presented as median (IQR) for the results. Differences between age, abstinence time, and BMI were compared using Student’s *t*-test. Differences between semen parameters were compared using Mann-Whitney U test. Differences between education level and occupation were compared using chi-square test and Fisher’s exact test, respectively

*BMI* body mass index, *SD *standard deviation, *IQR *interquartile range

^a^ Data regarding BMI were missing for 11 donors and 14 donors before and after the COVID-19, respectively. ^b^ Data regarding education level were missing for 14 donors and 1 donor before and after the COVID-19, respectively. ^c^ Data regarding occupation were missing for 14 donors and 1 donor before and after the COVID-19, respectively

We used linear regression analysis to eliminate the effects of covariates on semen quality of qualified sperm donors (Table 3). Model 1 adjusted for age. Model 2 adjusted for age and education level and model 3 adjusted for age, education level, and occupation. After adjusting for the above covariates, there were still no statistical differences in all semen parameters before and after the COVID-19 (all *P* > 0.05).

**Table 3:** Regression coefficients and 95% CIs of semen parameters of qualified sperm donors

	Model 1		Model 2		Model 3	
**Semen parameters**	*B*	95%CI	*B*	95%CI	*B*	95%CI
Volume (ml)	-0.008	-0.116, 0.100	0.009	-0.100, 0.117	0.007	-0.103, 0.117
Sperm concentration (10^6^/ml)	-0.072	-0.197, 0.053	-0.076	-0.203, 0.051	-0.078	-0.206, 0.050
Total sperm count (10^6^)	-0.080	-0.255, 0.095	-0.067	-0.245, 0.110	-0.071	-0.250, 0.108
Progressive motility (%)	0.014	-0.045, 0.073	0.016	-0.044, 0.076	0.012	-0.048, 0.072
Total motility (%)	0.002	-0.042, 0.047	0.005	-0.041, 0.050	0.002	-0.044, 0.047
Number of progressive motility (10^6^)	-0.066	-0.273, 0.141	-0.051	-0.261, 0.159	-0.059	-0.271, 0.152
Normal sperm morphology (%)	-0.023	-0.063, 0.017	-0.020	-0.060, 0.020	-0.021	-0.062, 0.020

Multiple linear regression was used to investigate the association between COVID-19 pandemic and semen quality. Model 1 adjusted for age. Model 2 adjusted for age and education level. Model 3 adjusted for age, education level, and occupation. *B*: beta-coefficient, 95% CI: 95% confidence interval

## Discussion

Our results showed no significant differences in semen parameters, except semen volume of candidate sperm donors, which increased after the outbreak. Interestingly, the average age of candidate and qualified sperm donors was higher after the outbreak, and education level and occupation of qualified sperm donors changed significantly after the outbreak. Most qualified sperm donors were students before the COVID-19 pandemic. After the outbreak, most university students did not return to school and stayed home, as the Chinese government took effective prevention and control measures to reduce coronavirus transmission [16]. Furthermore, due to the COVID-19 pandemic, many factories suspended production; therefore, many physical laborers had the time and energy to donate semen. This clearly changed the occupation, education level, and average age of sperm donors. More healthy young men, such as physical laborers, should be encouraged to be sperm donors to meet the increasing demand for donor sperm in the field of reproductive medicine.

In our study, the average age of qualified sperm donors was higher after the COVID-19. However, the effects of male aging on fertility remains uncertain and the review of the literature shows inconsistent results [17]. Previous studies showed that male aging was associated with a decline in fertility, as reflected by decreased pregnancy rates and increased time to pregnancy and frequency of subfecundity [18, 19]. Demir et al. [20] found that men’s age was a critical factor for intrauterine insemination (IUI) success, even if they had normal semen parameters. The association between age and sperm DNA damage was observed for several years. Aging men showed a significantly higher percentage of sperm DNA fragmentation (SDF) [21, 22]. Although the average age of qualified sperm donors increased after the COVID-19, it was still less than 30 years old, which is below the natural biological threshold for optimal male fertility potential that most researches have shown [23].

We also found that the proportion of qualified sperm donors with lower education levels increased significantly after the COVID-19 outbreak. Studies found that parental educational attainment was associated with offspring’s health, personality, and innate immune system regulation [24–26]. Both genetics and the early living environment of children account for these associations, but sperm donors do not have an impact on the living environment of their offspring. It is uncertain whether the development of offspring is affected by the educational attainment of sperm donors. It is necessary for human sperm banks to be aware of sociodemographic changes among sperm donors after the COVID-19 pandemic.

Although studies reported that people experienced great psychological stress and lifestyle changes that adversely affect semen quality [9–11] during the COVID-19 pandemic, no decline in semen quality of uninfected men was found. However, chronic stressors negatively affect both natural and specific immunity in humans [27]. Therefore, future studies with specific questionnaires and tests on immune system function, such as leukocyte numbers and levels of interferon-gamma (IFN-γ) [28], are warranted to shed more light on COVID-19 and reproductive health of uninfected men.

Research published since the COVID-19 outbreak has focused on the effects of COVID-19 on semen quality and hormone levels, but the results are controversial [29, 30]. According to a multicenter study, the overall andrological health of COVID-19 patients does not seem to be compromised 3 months after recovery [31]. We focused on semen quality of uninfected men after the COVID-19 outbreak by comparing the semen parameters of uninfected sperm donors. No decline in semen quality of sperm donors was observed in our study. In addition, available evidence showed that cryopreserved semen was free of SARS-CoV-2 and was safe for use [32, 33]. Therefore, there is no need for human sperm banks to consider the quality and safety of cryopreserved semen after the COVID-19 pandemic.

This study has some limitations. First, we lacked questionnaire data (e.g., psychological state and lifestyle changes) from sperm donors; therefore, we cannot provide strong evidence regarding the impact of the COVID-19 pandemic-related stress and lifestyle changes on semen quality of uninfected men. Second, the sperm donors were different donors before and after the COVID-19. Third, our findings may only illustrate the situation in one region of China because the influence of the COVID-19 pandemic may vary from place to place. Further studies are required to confirm our findings.

## Conclusion

No decline in semen quality of uninfected men was observed after the COVID-19 pandemic in Shandong, China. There is no concern about the quality and safety of cryopreserved semen in human sperm banks after the COVID-19 pandemic. In addition, the pandemic is constantly recurring and future studies with specific questionnaires and tests on immune system function are warranted to shed more light on COVID-19 and reproductive health of uninfected men.

## Data Availability

Data are available on reasonable request.

## Abbreviations

ACE2: angiotensin-converting enzyme 2
AID: artificial insemination with donor sperm
BMI: body mass index
IFN: interferon
IQR: interquartile range
IUI: intrauterine insemination
SARS-CoV-2: severe acute respiratory syndrome coronavirus 2
SD: standard deviation
SDF: sperm DNA fragmentation
